# Germination Characteristics Associated With Glutathione S-Transferases Endowed Quizalofop-p-Ethyl Resistance in *Polypogon fugax*

**DOI:** 10.3389/fpls.2022.861056

**Published:** 2022-05-18

**Authors:** Wen Chen, Yajun Peng, Qiaojiao Lin, Tianzhu Zhang, Bei Yan, Lianyang Bai, Lang Pan

**Affiliations:** ^1^College of Plant Protection, Hunan Agricultural University, Changsha, China; ^2^Plant Protection Institute, Hunan Academy of Agricultural Sciences, Changsha, China; ^3^Shandong Cynda Chemical Co., Ltd., Jinan, China; ^4^Hunan Agricultural Biotechnology Research Institute, Hunan Academy of Agricultural Sciences, Changsha, China

**Keywords:** *Polypogon fugax*, salt stress, osmotic potential, germination speed, herbicide resistance

## Abstract

Quantification of germination characteristics between herbicide-resistant and -susceptible weeds might provide methods to control resistant weeds and permit better prediction of evolution and persistence of herbicide resistance. This study aimed to compare the germination characteristics of Asian minor bluegrass (*Polypogon fugax*) populations that are resistant or susceptible to quizalofop-p-ethyl under controlled conditions, which the resistance mechanism is involved in glutathione S-transferases (GSTs) metabolism-based resistance. No major differences in seed germination were found at diverse temperatures, pH ranges, and light conditions. However, a significant difference that seed response to a gradient of osmotic and salt stress between the resistant and susceptible *P. fugax* populations were found. Two stress response genes (*P5CS-1* and *CDPK-2*) in *P. fugax* were likely involved in germination rate as well as germination speed in response to these stresses. Subsequently, population verification demonstrated that *P5CS-1* and *CDPK-2* genes may be linked to the resistance mechanism. Additionally, the two genes play an important role in response to salt stress and osmotic stress as shown by transcript abundance after stress treatments. Our findings suggest that the variation of the germination characteristics in *P. fugax* associates with the presence of GST-endowed resistance mechanism.

## Introduction

Weeds are among the most challenging issues in agricultural production systems, competing with crops for light, water, and soil nutrients (Oerke and Dehne, 2004). If left uncontrolled, the average yield of soybean, wheat, cotton, maize, rice, and potatoes will lose an estimated 35% (Oerke, 2005). Germination is a critical stage in weed development. Successful weed establishment mainly depends on its ability to evolve suitable germination dynamics under diverse agricultural practices (Tang et al., 2015). Weed germination is usually the consequence of the collaborative effects of both the environment and genetic factors (Nonogaki et al., 2010). Temperature, light, pH, salinity, and osmotic stresses are among the major environmental factors that affect weed seed germination (Chachalis and Reddy, 2000; Koger et al., 2004; Tang et al., 2015).

As for genetic factors, weeds containing alleles conferring resistance to herbicide may show a fitness cost. This means the phenomenon where one gene controls multiple traits, where at least one of them is beneficial and at least one has adverse side effects (Purrington, 2000; Vila-Aiub et al., 2009; Délye et al., 2013). A known connection is reported between target-site resistance (TSR) to acetyl-CoA carboxylase (ACCase) inhibitors, conferred by ACCase gene mutations, and seed germination variation (Vila-Aiub et al., 2005b; Menchari et al., 2008; Wang et al., 2010; Owen et al., 2011). These TSR-related mutations may damage the normal functioning of the target site enzymes, leading to the direct pleiotropic changes of resistant alleles (Vila-Aiub et al., 2005a; Gundel et al., 2008). Conclusive evidence about the direct role of herbicide resistance alleles in the fitness cost is not available. Fitness costs of resistance alleles are also associated with non-target-site resistance in ACCase-inhibitors resistant ryegrass (*Lolium rigidum* Gaudin). The possible reason for this phenomenon was an indirect effect in which more resources are allocated to resistance-related enzymes (Vila-Aiub et al., 2005a). However, the genetic linkage of herbicide resistance and other traits, or coinstantaneous selection of herbicide resistance and other traits could not be ruled out (Vila-Aiub et al., 2005a; Maity et al., 2021).

Germination in weed seeds is expected to be a two-phase reaction, including testa rupture and endosperm rupture (Weitbrecht et al., 2011). Compared to the seeds of the ACCase inhibitor susceptible phenotype, it has been reported that germination at constant temperatures in the seeds of resistant phenotype was inhibited, and the reason was Leu1781 *ACCase* caused a co-dominant delay in germination (Vila-Aiub et al., 2005b; Délye et al., 2013). Extensive research toward germination variations has been carried out (Gundel et al., 2008; Maity et al., 2021), progress on revealing germination gene in weedy plant species in relation to herbicide resistance has been until now slow. Proline biosynthesis from glutamate is catalyzed by bifunctional Δ-1-pyrroline-5-carboxylate synthetase (P5CS) that yields pyrroline-5-carboxylate (P5C) in a two-phase process (Hu et al., 1992), and elimination of P5CS feed-back inhibition protected plants from osmotic stress. Calcium ions (Ca^2+^) are related to signal transduction in answer to diverse environmental factors and Ca^2+^-dependent protein kinases (CDPKs) mainly help the plants to adjust to environments (Kudla et al., 2010). The serine/threonine protein kinase (STPK) is the foremost variety of protein kinase, which can help plant to regulate various biological processes to adjust to different environments (Alderwick et al., 2006). However, only a few P5CS, CDPK, and STPK genes have been characterized in the plants. In view of the importance of weed seed germination, determining the possible causes of germination deserves further study on these genes.

An ACCase inhibitor quizalofop-p-ethyl is widely used and effective for weed control in China. However, its extensive and continuous use has decreased quizalofop-p-ethyl sensitivity in weeds. Asian minor bluegrass (*Polypogon fugax*), as the annual winter grass, has become a predominant weed (Akopian and Ghukasyan, 2018). Quizalofop-p-ethyl resistant *P. fugax* now also occurs in several regions of China (Tang et al., 2014; Chen et al., 2020). Recently, we reported that resistance to quizalofop-p-ethyl in *P. fugax* was caused by non-target-site based involving glutathione S-transferases (GST; Chen et al., 2020). However, germination traits that could be related to the GST-endowed resistance mechanism have been little studied. It is vital that the selective resistance and sensitivity of weeds were investigated to elucidate potential associations between germination traits and herbicide resistance. These informations were useful for predicting the spread of resistance genes and determining the gene frequency in different environment, and also providing effective methods for controlling resistant *P. fugax* biotypes, as same as to investigate the germination biology (Vila-Aiub et al., 2005b, 2009). Thus, the present study aimed to (1) determine the effects of temperature, light, pH, osmotic and salt stress on *P. fugax* seed germination; (2) compare the difference in seed germination between the sensitive (S) and GST-endowed resistant (R) biotypes in response to the different environmental conditions; and (3) elucidate whether stress response genes are involved in *P. fugax* seed germination.

## Materials and Methods

### Seed Source and Identification of Biotypes

All seeds used in the experiment were originally from our previous research (Chen et al., 2020). The resistant *P. fugax* population (R) was collected from a winter canola fields (29.86°N, 103.85°E) in Qingshen county of Meishan, Sichuan, China. The susceptible *P. fugax* population (S) was collected from an uncultivated land (29.84°N, 103.85°E, about 2.6 km from the R population) where had never been applicated by quizalofop-p-ethyl.

A total of 50 plants from the R population were cultivated in 3-inch pots at constant temperature 15°C under a photoperiod of 12 h/12 h. When they grow to 3–4 leave stage, quizalofop-p-ethyl at the recommended label rate (67.5 g ai·ha^−1^) which was sufficient to control sensitive individuals was sprayed on the plants. Alive plants were pollinated to produce progeny populations in individual bags. Before the germination experiment, the seeds of resistant individual plants were pooled, and the susceptibility to quizalofop-p-ethyl was tested on the seedlings cultivated from these seeds. All plants (100 out of 100 plants) survived at the recommended label rate of quizalofop-p-ethyl. The seeds produced by these plants were stocked in envelopes at −20°C until use. The grain-filling seeds were selected and used in the following experiments.

### General Germination Test

Except where otherwise stated, 40 seeds were sown on petri-dish containing two pieces of filter paper soaking with 5 ml pH = 6.8 distilled water or the test solutions. The seeds then were transferred to the artificial weather educates box (Shanghai CIMO Medical Instrument Manufacturing Co., LTD, Shanghai, China) with the fluorescent lamps producing a photo-synthetic photon flux density of 140 μmol m^−2^ s^−1^. Seeds were incubated at a constant temperature of 15°C. Seed germination was monitored and recorded every 12 h for 21 days. Germinated seeds, in which the radicle was observed, were removed from the petri dish. Twenty-one days after the start of the experiment, 0.4% tetrazolium chloride solution was used to test the viability of the ungerminated seeds (ISTA, 1985), seeds showing a pink to reddish color were considered viable after soaking for 4 h. Seeds evaluated as viable were also recorded.

### Salt Stress Conditions for Germination Assays

The sodium chloride (NaCl) solutions were prepared to simulate salt stress. The effects of salt stress on *P. fugax* seed germination were determined using seven concentrations, including 0, 20, 40, 60, 80, 100, 120, and 140 mM. All the other procedures and conditions were performed as described above in the General germination test.

### Osmotic Stress Conditions for Germination Assays

The aqueous solutions with osmotic potentials were prepared, using poly ethylene glycol (PEG) 6000 (Solarbio, Beijing, China). The effects of osmotic stress on *P. fugax* seed germination were determined using eight treatments, including 0, −0.1, −0.2, −0.3, −0.4, −0.5, −0.6, and −0.7 MPa were prepared by dissolving 0, 67.2, 105.6, 135.7,161.3, 183.9, 204.5, and 223.4g PEG 6000 in 1 L of distilled water, respectively (Michel and Kaufmann, 1973). A constant temperature of 15°C was maintained in all growth incubators for these experiments. All the other procedures and conditions were performed as described above in the General germination test.

### Temperature Conditions for Germination Assays

Based on previous research (Tang et al., 2015) and according to the local climate, five constant temperature regimes (10, 15, 20, 25, and 30°C) and three fluctuating temperature regimes (20/15, 25/20, and 30/25°C day/night) in a 12-h photoperiod were selected to determine temperature effects on the germination of the two *P. fugax* populations. All the other procedures and conditions were performed as described above in the General germination test.

### Light Exposure Conditions for Germination Assays

Seed germination was examined under 12/12, 0/24, 24/0 h light/dark regimes per 24-h cycle. Two layers of tinfoil were used to wrap the dishes for dark treatment, and other treatments were unwrapped to allow for continuous lighting. Temperature condition was changed to 20°C (daytime, 12 h) and 15°C (night-time, 12 h) due to the faster germination speed detected under this condition. All the other procedures and conditions were performed as described above in the General germination test.

### The pH Conditions for Germination Assays

Seven treatments were conducted to determine the effects of pH on seed germination. Seeds from the two *P. fugax* populations were incubated in buffered solutions with pH 4, 5, 6, 7, 8, and 9, respectively, which were prepared according to the method described by Chachalis and Reddy (2000). A 2 mM potassium hydrogen phthalate buffer solution was adjusted to pH 4 with 1 M HCl. A 2 mM solution of MES [2-(N-morpholino) ethanesulfonic acid] was adjusted to pH 5 or 6 with 1 M NaOH. A 2 mM solution of HEPES [N-(2-hydroxymethyl) piperazine-N-(2-ethanesulfonic acid)] was adjusted to pH 7 or 8 with 1 M NaOH. A pH 9 buffer was prepared with 2 mM tricine [N-tris(hydroxymethyl) methylglycine] and adjusted with 1 M NaOH. Deionized water (pH 6.8) was used as a control. The pH values of the solution were calibrated using a pH meter (Sartorius Scientific Instrument Co., LTD, Beijing, China). Temperature condition was changed as described above in the Light exposure conditions for germination assays. All the other procedures and conditions were performed as described above in the General germination test.

### Statistical Analyses

Each treatment included four biological replicates in each germination experiment group and each replicate was assigned 40 seeds completely at random. Each experiment was repeated at least twice in which salt stress and osmotic stress conditions for germination assays were repeated three times. ANOVA analysis was performed to confirm that trial-by-treatment interactions were not significant (*p* > 0.05). Therefore, we pooled for the data for final analysis. The SPSS software (version 22.0, SPSS Inc., Chicago, IL) for a Tukey’s honestly significant test (*p* ≤ 0.05) was used to analyze the data for significance. The time-event model is used to analyze the data as described (Ritz et al., 2013).

Cumulative seed germination values for each biotype were fitted to a functional three-parameter sigmoidal model using least-squares non-linear regression (SigmaPlot 13.0, Systat Software Inc., San Jose, CA, United States). The model used was:


(1) $$E\;\left(\%\right)=a/\left(1+\exp\;\left(-\left(x-t_{G50}\right)/b\right)\right)$$

where *E* (%) is the cumulative germination over time (*x*); a is the maximum germination (%); *t*_G50_ is the time it takes to reach 50% of the final seed germination; and *b* indicates the slope around *t*_G50_. Parameter *b* provides an indication of the distribution of the response over time (i.e., low *b*-values indicate that a high proportion of the population germinates around *t*_G50_).

The germination indexes (GI, respectively) were calculated as described by the Association of Official Seed Analysis (1990) using the following formula:


(2) $$\mathrm{GI}=\frac{\mathrm{No}\;\mathrm{of germinated}}{d\;\mathrm{of first count}}+\ldots +\frac{\mathrm{No}\;\mathrm{of germinated}\;}{d\;\mathrm{of final count}}\;$$

The percentage of germination values at different osmotic potentials or salt concentrations were fitted to a functional three-parameter logistic model using SigmaPlot (Version 13.0, Systat Software Inc., San Jose, CA, United States). The model fitted was:


(3) $$G\;\left(\%\right)=G_{\max}/\left[1+\left(x/x50\right)\;g\right]$$

In this equation, *G* (%) represents the total germination (%) at NaCl concentration or osmotic potential *x*; *G*_max_ is the maximum germination (%); *x*50 is the NaCl concentration or osmotic potential for 50% inhibition of the maximum germination; and g indicates the slope.

### Gene Expression Analysis of Stress Response Genes in *Polypogon fugax*

Ten plants in each *P. fugax* population were cultivated to the 3–4-leaf stage under the artificial weather educates box above. Total RNAs were extracted and cDNAs were synthesized in each population following manufacturer protocol using RNAiso Plus (TaKaRa Biotech, China) and using HiScript II 1st Strand cDNA Synthesis Kit (Vazyme biotech, United States), respectively. *Elongation factor-1 alpha* (*EF-1*) was used as internal control genes (Chen et al., 2020). Due to lack of genomic data of *P. fugax*, a homologous cloning method was used to clone the conserved parts of *P. fugax* response genes. Based on the known P5CS, CDPK and STPK genes of rice (*Oryza sativa* L.), wheat (*Triticum aestivum* L.), mouseear cress [*Arabidopsis thaliana* (L.) Heynh.], and purple false brome [*Brachypodium distachyon* (L.) P. Beauv.], the conserved parts of *P. fugax* response genes were cloned, sequenced and blasted for confirmation. Specific quantitative real-time PCR (qRT-PCR) primer for the P5CS, CDPK and STPK genes was designed and assessed to amplify a single specific PCR amplification and no PCR amplification in the negative control (replaced the primers with ddH_2_O). Primers used for gene expression detection are listed in Supplementary Table S1 and primer efficiencies of all studied genes were 90–110% (Supplementary Table S1). The relative expression of genes was calculated using the 2^−ΔΔ*C*T^ method (Pan et al., 2016). Each experiment included three biological replications and repeated at least twice. Significant differences in the expression levels were analyzed using Welch’s *t*-test (Pan et al., 2016). In order to enhance data reliability, a higher change (3-fold) and *t*-test (*p* < 0.05) were used to determine up or down-regulation.

### Population Validation of Stress Response Genes and Germination Characteristics

To reveal whether the germination variations were linked to herbicide resistance, additional four quizalofop-p-ethyl-resistant and three quizalofop-p-ethyl-sensitive *P. fugax* populations were used for further validation. The collection sites of these populations were irregularly distributed, and they were separated by 2.6–700 km (Supplementary Table S2). Preliminary pot experiments revealed that these populations by NBD-CI pre-treatmented has no obvious visual effects on the quizalofop-p-ethyl susceptibility (unpublished data). The expression level of *P5CS-1*, *CDPK-2*, and *STPK-2* genes in these populations was measured using qRT-PCR, and compared to expression level of the same genes in the R population. Plant material cultivation, RNA extraction, cDNAs synthesis and gene expression measurements were as described above. Each experiment included three biological replications with 10 plants per replicate.

The germination characteristics of additional R and S populations were compared under salt (60 mM NaCl solutions) and osmotic (−0.2 MPa potentials) stress. Seeds were incubated with constant temperature of 15°C. Seed germination was monitored every 12 h for 14 days. All the other procedures and conditions were performed as described above.

### Expression Levels of Stress Response Genes Under Salt Stress and Osmotic Stress Treatment

To investigate whether *P5CS-1* and *CDPK-2* genes play a role in stress response, their expression levels were compared between the R and S *P. fugax* populations. At 3–4 the leaf stage, *P. fugax* seedlings were treated. For salt or osmotic stress, the plants were watered with 500 ml of 500 mM NaCl or 500 ml of 12.87% PEG (−0.3 MPa potentials), respectively. The aboveground parts of plants were collected at 0, 6, 12, and 24 h after stress treatment. RNA extraction, cDNAs synthesis and gene expression measurements were as described above. Each treatment included three biological replications with 10 plants per replicate.

## Results

### Effect of Salt Stress Conditions

**Figure 1:**
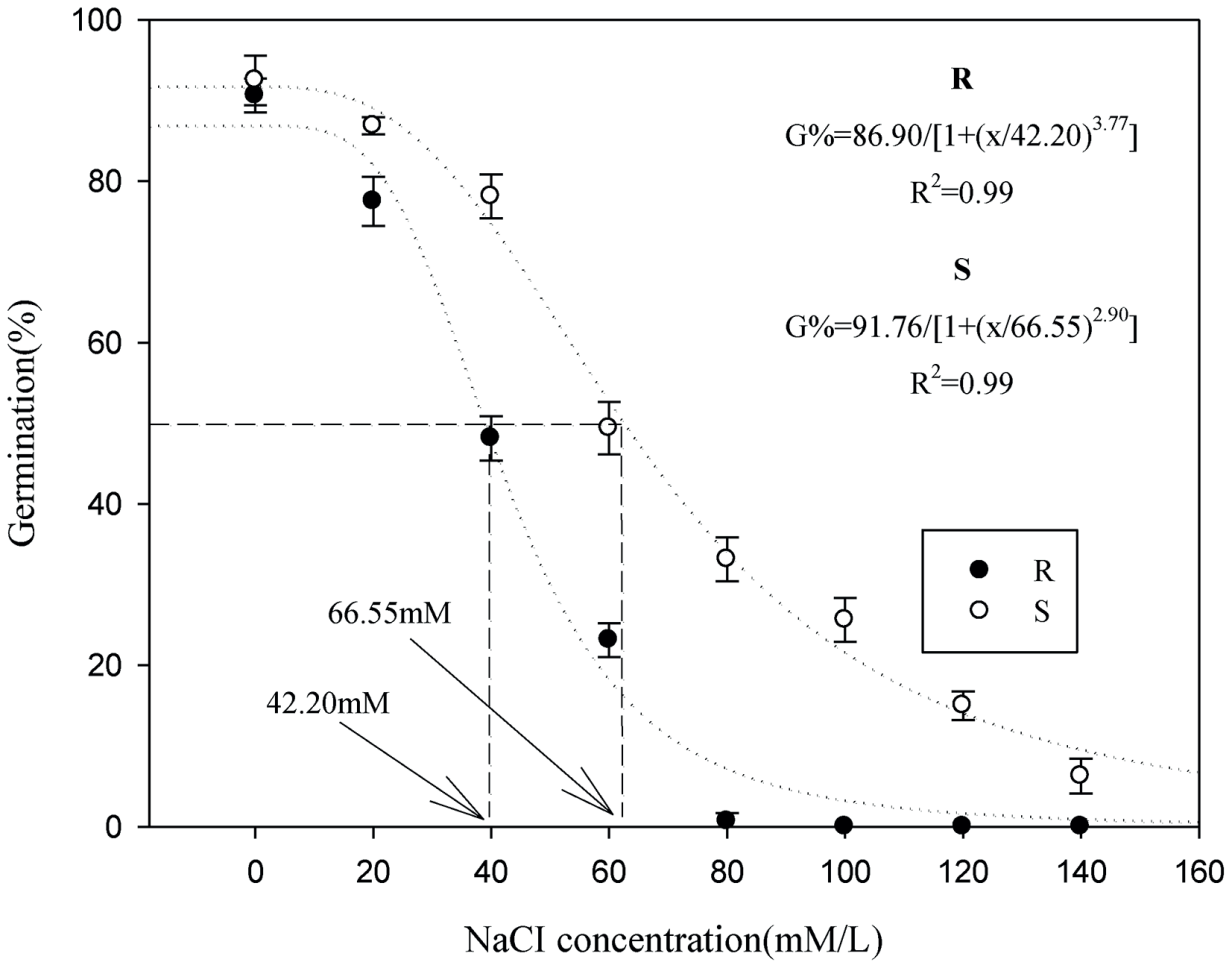
Effect of salt stress on the germination of the different *Polypogon fugax* populations. Error bars represent standard error of the means. Values represent the mean of eight replications with 40 seeds per replication. The abbreviations R and S represent for the resistant and sensitive *P. fugax* populations, respectively.

When the salt stress potential was increased from 0 to 140 mM, seed germination in the *P. fugax* populations decreased sharply (Table 1). The final germination rate of the S population (33–86%) was higher than that of the R population (0–77%, respectively; *p* < 0.05; Table 1), when the salt stress was increased from 20 to 80 mM. At 100–140 mM, the final germination rate of the S population (6–25%) was considerably higher, whereas no germination in the R population (0%) occurred under salt stress at more than 100 mM (Table 1). At more than 80 mM, there is no need for the *t*_G50_ of R population as no germination was detected (Table 1). At 20–80 mM, the *t*_G50_ of the R population was significantly higher than that of the S population (*p* < 0.05, Table 1). A three-parameter logistic model was fitted to the germination rate (%) of *P. fugax* obtained at different salt concentrations. The germination of the R and S population was half of the maximum germination when the salt concentrations were 42.20 and 66.55 mM, respectively (Figure 1).

**Table 1:** Effect of salt stress on the final germination rate, time to 50% germination (*t*_G50_), and germination index (GI) of *Polypogon fugax*.

Salt stress	The final germination rate ± SE (%)		Time to 50% germination (*t*_G50_) (days) ± SE		Germination index (GI) ± SE	
	R	S	R	S	R	S
0	90.6 ± 2.1a	92.5 ± 3.1a	7.70 ± 0.17a	8.15 ± 0.22ab	4.55 ± 0.16a	4.42 ± 0.11a
20 mM	77.5 ± 3.1b	86.9 ± 1.1a	8.59 ± 0.10b	7.60 ± 0.17a	3.51 ± 0.12c	4.26 ± 0.22a
40 mM	48.1 ± 2.7c	78.1 ± 2.7b	12.31 ± 0.15d	7.63 ± 0.34a	1.50 ± 0.08e	3.87 ± 0.08b
60 mM	23.1 ± 2.1e	49.4 ± 3.2c	12.87 ± 0.18de	7.84 ± 0.04a	0.69 ± 0.06fg	2.23 ± 0.13d
80 mM	0.6 ± 0.1g	33.1 ± 2.7d	NGg	8.95 ± 0.21b	NGi	1.46 ± 0.12e
100 mM	0g	25.6 ± 2.7e	NGg	11.00 ± 0.24c	NGi	0.93 ± 0.09f
120 mM	0g	15.0 ± 1.8f	NGg	13.00 ± 0.51e	NGi	0.45 ± 0.06gh
140 mM	0g	6.3 ± 2.2g	NGg	13.82 ± 0.41f	NGi	0.17 ± 0.06hi

SE, Standard error; NG, No germination. For each parameter, means within the columns for different biotypes followed by the same letter are not significantly different according to Tukey’s honestly significant test at *α* = 0.05.

### Effect of Osmotic Stress Conditions

**Figure 2:**
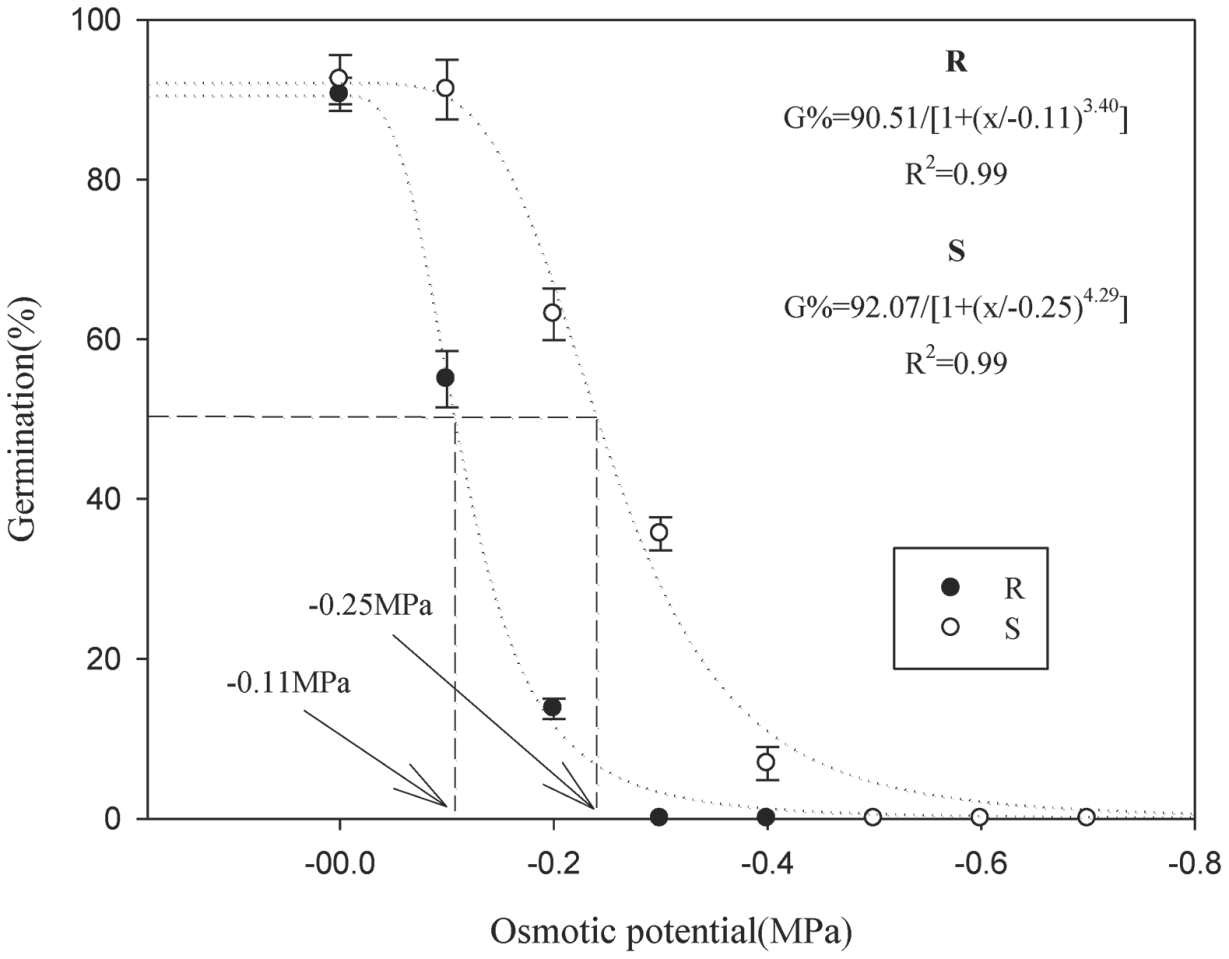
Effect of osmotic stress on the germination of the different *Polypogon fugax* populations. Error bars represent standard error of the means. Values represent the mean of eight replications with 40 seeds per replication. The abbreviations R and S represent for the resistant and sensitive *P. fugax* populations, respectively.

Seed germination in the R and S *P. fugax* populations decreased sharply when the osmotic potential was reduced from 0 to −0.4 MPa (Table 2). When the osmotic potential was reduced to −0.1 and −0.2 MPa, the final germination rate of the S population (91.2 and 63.1%, respectively) was higher than that of the R population (55.0 and 13.8%, respectively; *p* < 0.05; Table 2). In addition, no germination in the R population occurred at osmotic stress of less than −0.3 MPa, while that occurred at less than −0.5 MPa in the S population (Table 2). At more than −0.3 MPa, the *t*_G50_ of the R population was significantly higher than that of the S population (*p* < 0.05; Table 2). A three-parameter logistic model was fitted to the germination rate (%) of *P. fugax* obtained at different osmotic concentrations. The germination of the R and S population was half of the maximum germination when the osmotic concentrations were −0.11 and −0.25 MPa, respectively (Figure 2).

**Table 2:** Effect of osmotic stress on the final germination rate, time to 50% germination (*t*_G50_), and germination index (GI) of *Polypogon fugax*.

Osmotic stress	The final germination rate ± SE (%)		Time to 50% germination (*t*_G50_) (days) ± SE		Germination index (GI) ± SE	
	R	S	R	S	R	S
CK	90.6 ± 2.1a	92.5 ± 3.1a	7.70 ± 0.17a	8.15 ± 0.22ab	4.55 ± 0.16a	4.42 ± 0.11a
−0.1 Mpa	55.0 ± 3.5c	91.2 ± 3.8a	13.29 ± 0.22d	8.41 ± 0.10b	1.63 ± 0.05d	4.12 ± 0.17b
−0.2 Mpa	13.8 ± 1.3e	63.1 ± 3.2b	13.86 ± 0.15de	11.70 ± 0.43c	0.39 ± 0.03fg	2.20 ± 0.10c
−0.3 Mpa	0e	35.6 ± 2.1d	NGg	14.19 ± 0.18e	NGg	1.04 ± 0.06e
−0.4 Mpa	0e	6.9 ± 2.1e	NGg	16.97 ± 0.35f	NGg	0.16 ± 0.05g

SE, Standard error; NG, No germination. For each parameter, means within the columns for different biotypes (R and S *Polypogon fugax* populations) followed by the same letter are not significantly different according to Tukey’s honestly significant test at *α* = 0.05.

### The R/S *Polypogon fugax* Populations Are Insensitive to Changes of Temperature Conditions

**Figure 3:**
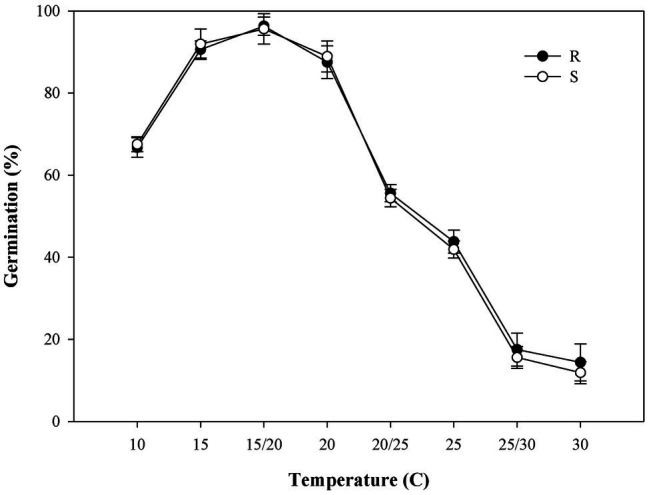
Effect of temperature on the final germination rate of the different *Polypogon fugax* populations. Error bars represent standard error of the means. Values represent the mean of eight replications with 40 seeds per replication. The abbreviations R and S represent for the resistant and sensitive *P. fugax* populations, respectively.

Both the R/S *P. fugax* populations had more than 90% final germination rate under temperature 15°C and 15/20°C tested with no significant difference (*p* > 0.05; Figure 3), however, 15/20°C was more suitable for *P. fugax* germination duo to the lower *t*_G50_ values and higher GI (Supplementary Table S3). Sigmoidal models were used to test *t*_G50_, confirming the time (days) required to achieve 50% germination. As expected, at warmer temperatures, all populations germinated more rapidly, as indicated by the lower *t*_G50_ values (Supplementary Table S3). Notably, there was no significant difference among the S and R populations for *t*_G50_ and GI at all temperatures investigated (*p* > 0.05; Supplementary Table S3).

### Different Light Exposure Conditions Did Not Differentially Affect the Germination of the R/S *Polypogon fugax* Populations

**Figure 4:**
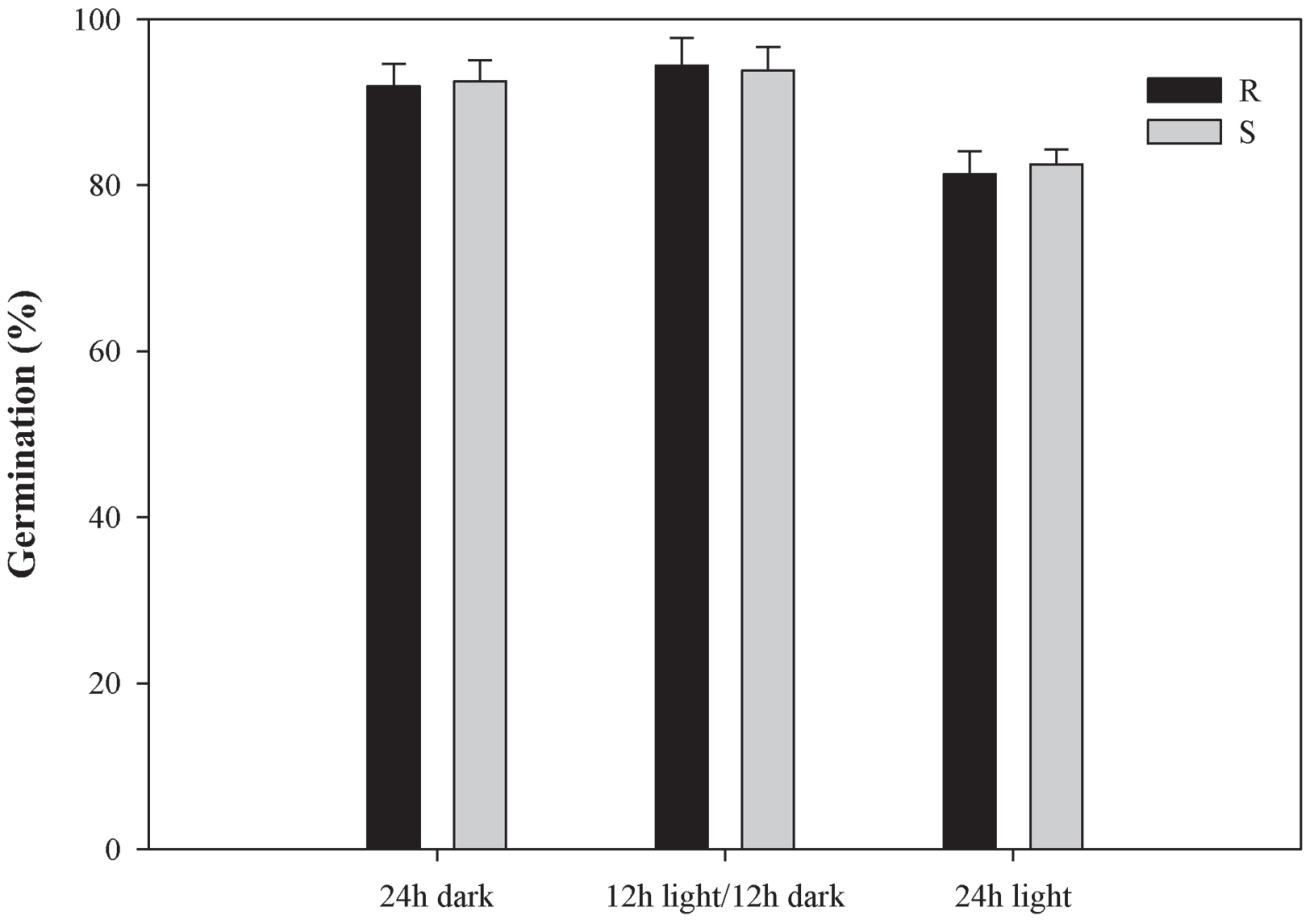
Effect of light on the final germination rate of the different *Polypogon fugax* populations. Error bars represent standard error of the means. Values represent the mean of eight replications with 40 seeds per replication. The abbreviations R and S represent for the resistant and sensitive *P. fugax* populations, respectively.

Exposure to light had no effect on the germination of most seeds in the *P. fugax* populations (Figure 4). The germination rate for *P. fugax* populations exposed to a 24-h photoperiod was significantly lower than that for other two light exposure conditions (*p* < 0.05; Figure 4; Supplementary Table S4). No difference in germination rate was found under light/dark conditions and under dark conditions alone for most seeds in the *P. fugax* populations (Figure 4). The *t*_G50_ value and GI of the R population were similar to those of the S population (*p* > 0.05; Supplementary Table S4).

### Changes in pH Did Not Differentially Affect the Germination of R/S *Polypogon fugax* Populations

**Figure 5:**
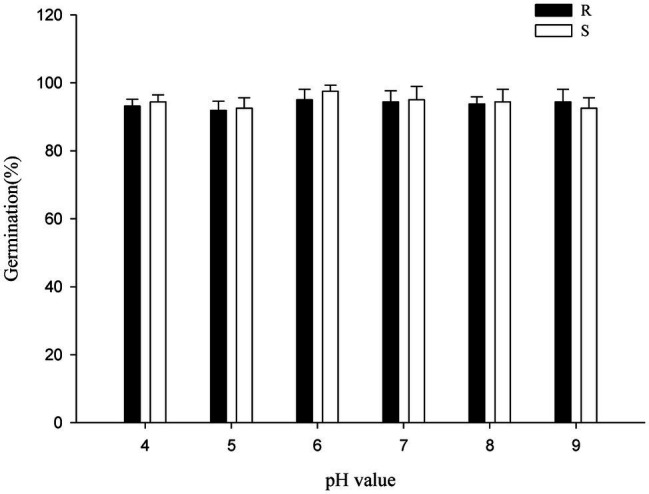
Effect of pH on the germination of the different *Polypogon fugax* populations. Error bars represent standard error of the means. Values represent the mean of eight replications with 40 seeds per replication. The abbreviations R and S represent for the resistant and sensitive *P. fugax* populations, respectively.

The *P. fugax* populations were insensitive to pH changes. Compared with the control (distilled water), the germination of the *P. fugax* populations was not influenced by pH between 4 and 9 (Figure 5). All the biotypes had similar germination rates of above 90% under all pH conditions (*p* > 0.05; Figure 5), similar to other weed species such as Asian Minor bluegrass (Tang et al., 2015), Italian ryegrass (Nandula et al., 2009), and jointed goatgrass (Fandrich and Mallory-Smith, 2006). The oilseed rape fields in regions of Sichuan Province, from where *P. fugax* were collected, have soil pH ranging from 4 to 9, like those in Anhui and Jiangsu Provinces.

### The Expressions Level of Several Stress Response Genes in R and S *Polypogon fugax*

**Figure 6:**
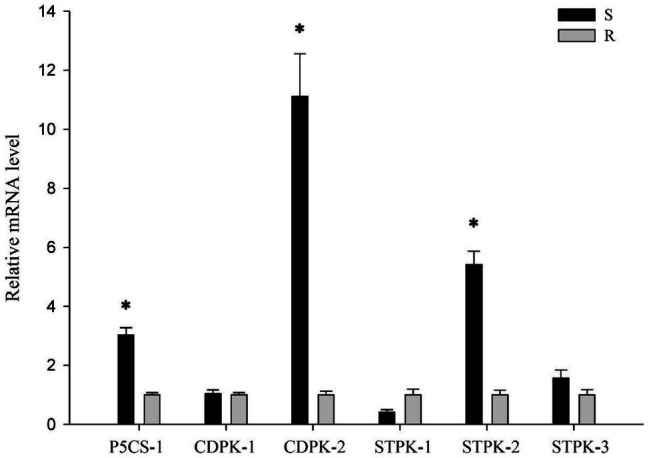
The relative expression level of stress response genes in the R and S *Polypogon fugax* populations using quantitative reverse-transcription polymerase chain reaction (qRT-PCR). The qRT-PCR was performed using three independent biological replicates and repeated at least twice (two technical replicates). Data are the mean values of six biological replicates. The standard errors of the means are described by vertical bars. The abbreviations R and S represent for the resistant and sensitive *P. fugax* populations, respectively. *Indicates significant differences (*p* = 0.05) by Turkey’s test.

To determine whether P5CS, CDPK and STPK genes are involved in the stress response of *P. fugax*, we isolated one P5CS, two CDPK and three STPK genes in *P. fugax*. The expressions of *CDPK-1*, *STPK-1*, and *STPK-3* did not change considerably between each population (Figure 6). In addition, the expressions of *P5CS-1*, *CDPK-2*, and *STPK-2* genes in the R population was about 3.03, 11.12, and 5.42 times lower than those in the S population, respectively (Figure 6). The expression patterns of these three genes in the *P. fugax* populations were consistent with the final germination rates in response to osmotic and salt stress, indicating that these genes might be involved in stress response during seed germination in *P. fugax*.

### *P5CS-1* and *CDPK-2* Genes Might Be Linked to the Resistance Mechanism

To illuminate whether *P5CS-1*, *CDPK-2*, and *STPK-2* genes are related to GST-involved resistance mechanism in *P. fugax*, the expression level of the three genes was measured in three quizalofop-p-ethyl-sensitive (S1, S2, and S3) and four quizalofop-p-ethyl-resistant (R1, R2, R3, and R4) populations with no GST-mediated resistance mechanism (Table 3). *P5CS-1* and *CDPK-2* genes were significantly up-regulated in seven *P. fugax* populations compared with the R population (Table 3). However, *STPK-2* gene was not up-regulated in most populations, with down-regulation only in one (Table 3). This result implies two possibilities: (1) *P5CS-1* and *CDPK-2* genes were constitutively and lowly expressed in the R population, indicating that these two stress response genes might be linked to the GST-endowed resistance mechanism; (2) it could not be ruled out that the high expression of the *STPK-2* gene conferred an enhanced ability to tolerate stress in the S population.

**Table 3:** The expression levels of stress response genes in seven different populations.

Population^a^	Fold change relative to the R population^b^		
	P5CS-1	CDPK-2	STPK-2
R1	4.65^*^	10.32^**^	1.08
R2	4.14^*^	15.84^*^	2.02
R3	2.49^*^	17.21^*^	0.42
R4	3.81^***^	20.06^**^	2.15
S1	3.51^*^	6.3^**^	1.13
S2	4.04^*^	5.07^**^	0.48^*^
S3	4.39^*^	6.09^**^	0.78

a R1-4 and S1-3 represented the additional four quizalofop-resistant and three quizalofop-sensitive populations, respectively. The seven populations were not affected on the sensitivity level of quizalofop-p-ethyl by the pretreatment of the GST inhibitor NBD-CI.

b Indicate significant differences between theses populations and the R population by Turkey’s test. *p*-Value of <0.05, 0.01, and 0.001 indicated by ^*^, ^**^, and ^***^, respectively.

In addition, the germination characteristics of these seven populations and the S population were characterized under salt stress and osmotic stress. Germination rate of the S3 and R3 populations was significantly higher and lower than that of S population, respectively, and there was no significant difference in other populations under salt stress (*p* > 0.05; Table 4). Under osmotic stress, germination rate of the R2 and R3 population was significantly higher than that of the S population, and there was no significant difference in other populations (Table 4). This result revealed compared with the R population, the S population with only the overexpression of the *STPK-2* gene did not show a specific ability to tolerate stress, refuting the second possibility mentioned above.

**Table 4:** Germination characteristics of seven different populations under salt and osmotic stress.

Population	Salt stress (60 mM)			Osmotic stress (−0.2 Mpa)		
	GR	*t*_G50_	GI	GR	*t*_G50_	GI
S	46.25 ± 2.80ab	6.59 ± 0.12a	2.65 ± 0.15ab	48.75 ± 2.17a	12.54 ± 0.39a	1.83 ± 0.09a
S1	43.13 ± 3.70b	6.72 ± 0.30a	2.41 ± 0.24bc	51.88 ± 4.80a	11.36 ± 0.58a	1.96 ± 0.20a
S2	44.38 ± 2.72b	6.61 ± 0.31a	2.57 ± 0.17bc	51.25 ± 5.15a	11.34 ± 0.49a	1.98 ± 0.30a
S3	52.50 ± 1.77a	6.57 ± 0.34a	3.06 ± 0.07a	53.13 ± 3.70a	14.07 ± 2.21a	2.04 ± 0.25a
R1	43.75 ± 2.28b	6.62 ± 0.26a	2.52 ± 0.20bc	54.38 ± 3.25a	12.73 ± 2.44a	2.17 ± 0.25a
R2	41.88 ± 2.07b	7.86 ± 0.30b	2.09 ± 0.15c	56.25 ± 5.73a	12.80 ± 2.29a	2.23 ± 0.28a
R3	40.63 ± 2.07b	7.29 ± 0.41ab	2.13 ± 0.18c	58.13 ± 2.72a	12.40 ± 1.36a	2.22 ± 0.13a
R4	43.75 ± 2.80b	6.56 ± 0.31a	2.57 ± 0.18bc	53.75 ± 5.73a	12.15 ± 0.51a	2.04 ± 0.16a

SE, Standard error. For each parameter, means within the columns for different biotypes (R and S *Polypogon fugax* populations) followed by the same letter are not significantly different according to Tukey’s honestly significant test at *α* = 0.05.

### Response Patterns of *P5CS-1* and *CDPK-2* Genes Under Salt Stress and Osmotic Stress

**Figure 7:**
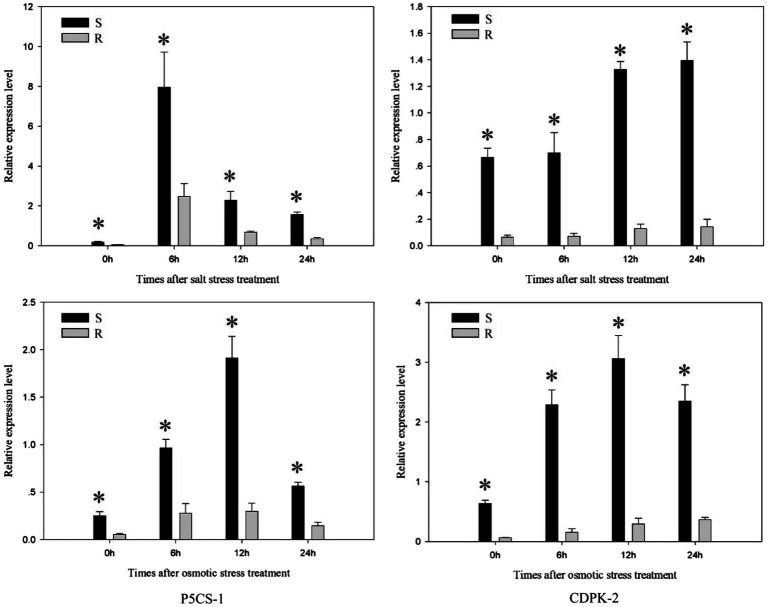
The expression level of *P5CS-1* and *CDPK-2* gene in the R and S populations after 0, 6, 12, and 24 h treatments of salt stress and osmotic stress, respectively. The standard errors of the means are described by vertical bars. The abbreviations R and S represent for the resistant and sensitive *Polypogon fugax* populations, respectively. *Indicates significant differences (*p* = 0.05) by Turkey’s test.

Further studies compared the expression changes of stress response genes under salt stress and osmotic stress. The expression level of *P5CS-1* gene in each population (the R and S populations) exhibited a clear up-regulation to the highest level and then decreased with treatment time (Figure 7). The expression of *P5CS-1* in the S population was 3.22–6.4 times (*p* < 0.05) higher than those in the R population at 0, 6, 12, and 24 h after salt stress and osmotic stress treatment. In response to salt stress and osmotic stress, *CDPK-2* gene in each population showed a continuous rise or first increase and then decline. The expression of *CDPK-2* gene in the R population was 4.12–14.7 times (*p* < 0.05) lower than those in the S population at 0, 6, 12, and 24 h after salt stress and osmotic stress treatment. This result implied that *P5CS-1* and *CDPK-2* genes play an important role in response to salt stress and osmotic stress.

## Discussion

In agricultural ecosystem, the seed germination time is one of the most important events that determine the success or failure of the propagation and invasion of annual plant species (Forcella et al., 2000). In the present study, we used weeds with characterized genotypes at a locus endowing herbicide resistance to assess the germination behavior.

Salt stress is an important factor that adversely affects germination of many weed species (Chachalis and Reddy, 2000; Singh et al., 2012). Our data suggested that the germination variations of R *P. fugax* population were manifested in the low seed germination rate and delayed germination under salt stress. However, compared to the S population, the germination variations may provide a greater opportunity for resistance evolution of the R population in modern agricultural systems. With the accelerated serious of soil salinization and the reduction of soil osmotic stress in China (Yu et al., 2010; Hussain et al., 2020), early-emerging tolerant weeds are largely controlled by pre-sowing management measures, whereas late-emerging weeds (due to delayed germination) are exposed to post-emergence herbicides, with opportunities for survival adaptation (Maity et al., 2021).

The well-watered soils are classified as those with an osmotic potential between 0 and −0.3 MPa (Haswell and Verslues, 2015). The final germination rates of all populations were less than 40% at osmotic stress of less than −0.3 Mpa, similar to the previous results (Wang et al., 2017). This indicated that the germination of *P. fugax* depends on soil moisture levels. Thus, future management should aim to utilise drought-tolerant crops and alter soil moisture levels to help compete with and suppress *P. fugax* germination. In addition, our findings might help explain the relationship between water availability and germination for *P. fugax* having GST-endowed resistance mechanism. A glyphosate-resistant Italian ryegrass biotype was shown to have a higher germination rate than its susceptible biotype (Lee et al., 2014). In the present study, discrepancy was found in the germination rate between the quizalofop-p-ethyl-resistant *P. fugax* and its susceptible population. However, the resistant *P. fugax* population with GST-endowed resistance showed different germination pattern with the susceptible population. The results of the final germination rate in each biotype (Table 2) indicated that this rate at different osmotic stress conditions of resistant *P. fugax* population might be linked to the resistance mechanism.

Germination consistently took place in all *P. fugax* populations tested across a wide range of temperature conditions (Figure 3; Supplementary Table S3), and germination under all light conditions (Figure 4), indicating that *P. fugax* do not require light for germination. In addition, our results indicated that all *P. fugax* populations can better adjust to this pH conditions, and that none of the population had a cost in germination for fitness over the wide pH range. In our results, the germination differences of R *P. fugax* population were manifested in the low seed germination rate and delayed germination under salt stress and osmotic stress compared with the S *P. fugax* population. Subsequently, the germination variation mechanism and its relationship with the resistance mechanism had been further studied.

Several studies have suggested the possible correlation between resistance to ACCase inhibitors and the changes in seed germination (Vila-Aiub et al., 2005b; Menchari et al., 2008; Owen et al., 2011); nevertheless, the genetic basis of this correlation has not yet been investigated. This study investigated the genetic basis for the correlation between germination rate of quizalofop-p-ethyl-resistant *P. fugax* and its susceptible population. Some P5CS, CDPK and STPK genes in other species were found to be regulated by heat or drought stress (Hu et al., 1992; Alderwick et al., 2006; Kudla et al., 2010). In the present study, the *P5CS-1* and *CDPK-2* genes showed distinctive response patterns under salt stress and osmotic stress. To our knowledge, this is the first study to identify these genes and analyze their expression profiles in *P. fugax*. Furthermore, the expression of *P5CS-1* and *CDPK-2* gene was lower in the R *P. fugax* population than in all other tested populations, consistent with previous findings on their low final germination rate under different osmotic and salt stress conditions. Some PSCS and CDPK genes were involved in the regulation of proline biosynthesis and the translation of phosphorylation signals, respectively, and both played a role in responding to abiotic stress in plants (Franz et al., 2011; Asano et al., 2012). Recent studies have shown that herbicides stress could induce changes in the transcription levels of *P5CS* and *CDPK* genes in plants (Boulahia et al., 2016; Wang et al., 2021; Rangani et al., 2022). It was reported that P5CS activity and its gene expression level were reduced by exogenous application of ethanolamine (Rajaeian et al., 2017). In our study, although resistance genes were continuously selected under herbicide stress, *P5CS-1* and *CDPK-2* genes may be negatively affected, resulting in the decrease of gene transcription levels. These results suggest that *P5CS-1* and *CDPK-2* genes are not only likely to be involved in stress tolerance during *P. fugax* seed germination, but also related to GST-mediated resistance mechanism.

In addition, P5CS and CDPK genes were found to be related to not only osmotic stress but also delayed germination (Hu et al., 1992; Kudla et al., 2010), indicating that these genes might also be related to the germination speed. Notably, in the present study, we found that the results of germination speed were consistent with the expression patterns of *P5CS-1* and *CDPK-2* genes in different *P. fugax* populations. Thus, these genes in *P. fugax* might be involved in not only response to stress during seed germination but also germination speed. Further evaluations (e.g., forward genetic validation, possible inhibitor identification, and progeny test) are necessary to validate this.

## Conclusion

In summary, our study showed the germination characteristics of GST-endowed resistant *P. fugax* population on seed germination under different environmental conditions. GST-endowed resistance mechanism in *P. fugax* has contrasting effects on seed germination and seed germination speed under different osmotic and salt stress. Our findings suggest that the variations in germination characteristics may depend on the resistance genes and plant genetic background. Although the specificity of germination in weed populations is very high and local diversities are common, this assumption still holds based on the existing results. Large-scale population verification experiments will be used for this study due to a lack of direct mechanistic approach in the future. This underlines the necessity to identify the resistance gene(s) present in a weed population before designing resistance-management strategies. The variation of germination characteristics according to the environment and weed population hampers the success of specific cultural control practices. A solution could be a weed control program maximizing the diversity of cultural practices and including anti-resistance cultural practices to avoid or reduce selection for resistant alleles.

## Data Availability Statement

The raw data supporting the conclusions of this article will be made available by the authors, without undue reservation.

## Author Contributions

WC, LB, and LP designed the experiments. WC, YP, QL, and BY performed the experiments. WC, TZ, and LP analyzed the data. WC, YP, and LP wrote and revised the manuscript. All authors contributed to the article and approved the submitted version.

## Funding

This research was financially supported by the National Natural Science Foundation of China (31901905 and 32001923), the Natural Science Foundation of Hunan Province, China (2021JJ20002 and 2019JJ50270), the Science and Technology Innovation Program of Hunan Province (2021RC3087), and China Agriculture Research System (CARS-16-E19).

## Conflict of Interest

TZ is employed by Shandong Cynda Chemical Co., Ltd.

The remaining authors declare that the research was conducted in the absence of any commercial or financial relationships that could be construed as a potential conflict of interest.

## Publisher’s Note

All claims expressed in this article are solely those of the authors and do not necessarily represent those of their affiliated organizations, or those of the publisher, the editors and the reviewers. Any product that may be evaluated in this article, or claim that may be made by its manufacturer, is not guaranteed or endorsed by the publisher.
