# Supplementary material for: Germination Characteristics Associated With Glutathione S-Transferases Endowed Quizalofop-p-Ethyl Resistance in Polypogon fugax
Source: Front Plant Sci. 2022 May 18;13:861056. doi: 10.3389/fpls.2022.861056 (PMC9158530; doi:10.3389/fpls.2022.861056)
Supplement: Supplementary file 1 [file Table_1.DOC]

**Supplementary** **Tables**

**Table S1.** Primers used for the amplification of *P. fugax* genes for the qRT-PCR analysis

| Gene | Direction | Sequence (5′–3′) | Amplicon size (bp) | Slope | R^2^ | EFF% |
| --- | --- | --- | --- | --- | --- | --- |
| UBQ | Forward | GGTTGACCCGAAGAAGTTCA | 179 | -3.51 | 0.996 | 92.91 |
|  | Reverse | TGCTGTTGAAACTGGAGACG |  |  |  |  |
| P5CS-1 | Forward | GCTCTAGCAATCCGAAGTGG | 242 | -3.39 | 0.983 | 97.08 |
|  | Reverse | TTCGTTGCTGCTTTGATTTG |  |  |  |  |
| CDPK-1 | Forward | GGAAACGCAGTGGTAATGTC | 242 | -3.12 | 0.957 | 109.01 |
|  | Reverse | GGGACAGGATACATCGAGAAAG |  |  |  |  |
| CDPK-2 | Forward | AGTCTTGGTGGTCGTTCTTG | 249 | -3.20 | 0.981 | 105.49 |
|  | Reverse | CCCCTAAAGCCAAGTACGTC |  |  |  |  |
| STPK-1 | Forward | TTCTGTCCCTTGGGTTGTTC | 204 | -3.15 | 0.914 | 107.56 |
|  | Reverse | GTGGACTCATCCCTGGCTAA |  |  |  |  |
| STPK-2 | Forward | TCTTGCACTTTTCCATGCTG | 166 | -3.47 | 0.969 | 94.20 |
|  | Reverse | ACCATGCACACCTGTCGTTA |  |  |  |  |
| STPK-3 | Forward | TTCGGTGTGATGATTTTGGA | 242 | -3.45 | 0.986 | 94.84 |
|  | Reverse | CTGATCGTTGACATGGTTGG |  |  |  |  |

**Table S2. Detailed collecting sites and locations of *Polypogon fugax*.**

| Biotypes | Collecting locations | Site | Latitude and longitude |
| --- | --- | --- | --- |
| R | Qingshen county of Meishan, Sichuan | winter canola fields | 29.86°N, 103.85°E |
| S | Qingshen county of Meishan, Sichuan | uncultivated lands | 29.84°N, 103.85°E |
| R1 | Dayi county of Chengdou, Sichuan | winter canola fields | 30.51°N, 103.63°E |
| R2 | Chongzhou City, Sichuan | winter canola fields | 30.68°N, 103.56°E |
| R3 | Pujiang county of Chengdou, Sichuan | winter canola fields | 30.17°N, 103.50°E |
| R4 | Chengguan County of Guiyang, Guizhou | winter canola fields | 27.11°N, 106.99°E |
| S1 | Qingshen county of Meishan, Sichuan | winter canola fields | 29.95°N, 103.78°E |
| S2 | Yaxi County of Zunyi, Guizhou | winter canola fields | 27.60°N, 106.73°E |
| S3 | Baiyun County of Anshun, Guizhou | winter canola fields | 26.37°N, 106.24°E |

**Table S3.** Effect of temperature on the final germination rate, time to 50% germination (*t*_G50_), and germination index (GI) of *Polypogon fugax*

| Temperature | The final germination rate ± SE (%) | | Time to 50% germination (*t*_G50_) (days)±SE | | Germination index (GI) ±SE | |
| --- | --- | --- | --- | --- | --- | --- |
|  | R | S | R | S | R | S |
| 10 °C | 66.7±2.4c | 67.5±1.8c | 14.36±0.12d | 14.38±0.17d | 1.76±0.07d | 1.79±0.05d |
| 15 °C | 90.6±2.1ab | 91.9±3.7ab | 7.11±0.40c | 7.06±0.24c | 5.07±0.26c | 5.03±0.34c |
| 15/20°C | 96.3±2.2a | 95.6±3.7ab | 3.52±0.15a | 3.80±0.08a | 10.02±0.37a | 9.21±0.37a |
| 20°C | 87.5±4.0b | 88.9±3.8ab | 5.10±0.39b | 4.80±0.63b | 6.61±0.29b | 7.19±0.77b |
| 20/25°C | 55.6±2.1d | 54.4±2.1d | 4.24±0.53ab | 4.26±0.61ab | 4.69±0.32c | 4.55±0.12c |
| 25°C | 43.8±2.8e | 41.9±2.1e | 3.54±0.17a | 3.59±0.24a | 4.11±0.43c | 4.13±0.23c |
| 25/30°C | 17.5±4.0f | 15.6±2.7f | 3.84±0.14a | 4.04±0.53a | 1.43±0.18d | 1.67±0.44d |
| 30°C | 14.4±4.5f | 11.9±2.7f | 3.34±0.33a | 3.66±0.58a | 1.48±0.48d | 1.19±0.16d |

SE: Standard error.

For each parameter, means within the columns for different biotypes (R and S *Polypogon fugax* populations) followed by the same letter are not significantly different according to Tukey's honestly significant test at α=0.05.

**Table S4.** Effect of light on the final germination rate, time to 50% germination (*t*_G50_), and germination index (GI) of *Polypogon fugax*

| Light | The final germination rate ± SE (%) | | Time to 50% germination (*t*_G50_) (days)±SE | | Germination index (GI) ±SE | |
| --- | --- | --- | --- | --- | --- | --- |
|  | R | S | R | S | R | S |
| 24h dark | 91.9±2.7a | 92.5±2.5a | 4.26±0.12c | 3.90±0.08bc | 7.86±0.31bc | 8.49±0.22b |
| 12hlight/12h dark | 94.4±3.3a | 93.8±2.8a | 3.32±0.15a | 3.70±0.18ab | 10.04±0.43a | 9.47±0.38a |
| 24h light | 81.3±2.8b | 82.5±1.8b | 4.04±0.25bc | 4.06±0.24bc | 7.27±0.21c | 7.51±0.28c |

SE: Standard error.

For each parameter, means within the columns for different biotypes (R and S *Polypogon fugax* populations) followed by the same letter are not significantly different according to Tukey's honestly significant test at α=0.05.
